# Circadian gene *Cry1* inhibits the tumorigenicity of hepatocellular carcinoma by the BAX/BCL2-mediated apoptosis pathway

**DOI:** 10.1515/biol-2025-1178

**Published:** 2025-10-13

**Authors:** Xuening Wu, Yilong Zhao, Yilin Wu, Leqing Li, Xinyu Guo, Sumeng Jiang, Qi Wang, Shujing Li, Yuanyuan Wang, Huanfeng Hao

**Affiliations:** School of Life Science, Bengbu Medical University, Bengbu, 233000, China; Anhui Engineering Research Center for Neural Regeneration Technology and Medical New Materials, Bengbu Medical University, Bengbu, 233000, China; Anhui Provincial Key Laboratory of Tumor Evolution and Intelligent Diagnosis and Treatment, Bengbu Medical University, Bengbu, 233000, China

**Keywords:** circadian rhythm, *Cry1*, hepatocellular carcinoma

## Abstract

Hepatocellular carcinoma (HCC) is a leading cause of cancer-related mortality worldwide, and emerging evidence implicates circadian rhythm disruption in its pathogenesis. Here, we identified the core circadian gene *Cryptochrome1* (*Cry1*) as a potential tumor suppressor in HCC. Clinical analysis revealed that low *Cry1* expression correlated with poor prognosis, showing a median survival of 36 vs 47 months, and *Cry1* expression was significantly reduced in HCC cell lines (0.6-fold in SMMC-7721 vs LO2). Functional studies demonstrated that *Cry1* overexpression reduced proliferation by 30% with more cells in the G1 phase, as well as inhibited migration/invasion, while *Cry1* knockdown increased proliferation by 50% with less cells in the G1 phase and increased migration/invasion. Finally, we found *Cry1* depletion downregulated pro-apoptotic BAX and upregulated anti-apoptotic BCL2, while *Cry1* overexpression produced the opposite effects, suggesting its role in apoptosis via the BCL2/BAX-mediated apoptosis pathway. These findings indicate that *Cry1* acts as a tumor suppressor in HCC, providing insights into the circadian dysfunction-cancer pathogenesis connection and its potential as a diagnostic biomarker and therapeutic target requires further verification through preclinical and clinical investigations in the future.

## 1 Introduction

Liver cancer accounts for 4.5% of global cancer incidence and 7.8% of cancer-related deaths, with higher incidence rates observed in low-income countries [1]. The most prevalent kind of liver cancer in the world is hepatocellular carcinoma (HCC), which constitutes the lion’s share of all liver cancer cases [2]. Globally, liver cancer is a leading cause of cancer-related mortality [3] and ranks among the top six most common malignancies in recent years [4]. Moreover, the prognosis for liver cancer patients is poor, with only 5–15% eligible for surgical intervention [5] – an option limited to early-stage patients without cirrhosis [1]. Despite recent advances in treatment, the prognosis for HCC remains poor, as patients are often diagnosed at advanced stages [6]. Furthermore, both chemotherapy and targeted therapies are associated with significant side effects [7]. Therefore, identifying novel biomarkers for diagnosis and treatment, along with elucidating underlying mechanisms, is essential for the early detection and effective management of HCC.

Circadian rhythms are endogenous ∼24 h cycles observed in a wide range of organisms, including plants, mammals, fungi, and bacteria. These rhythms help organisms adapt to periodic environmental changes such as fluctuations in light and temperature [8,9]. At the molecular level, circadian clocks are regulated by transcriptional and translational feedback loops [10]. In mammals, the transcription of *PERIOD (PER)* and *CRYPTOCHROME (CRY)* is activated by the heterodimeric transcription factors BMAL1 and CLOCK, which bind to E-box elements in their promoter regions. PER and CRY proteins then accumulate in the cytoplasm, form complexes, translocate into the nucleus, and inhibit BMAL1–CLOCK-mediated transcription [11]. Disruption of circadian rhythms has become increasingly common and is linked to various diseases in epidemiological studies [12]. In particular, dysregulation of circadian genes is closely associated with cancer progression [13]. Recent studies show that the expression patterns of clock-controlled genes and core clock genes are disrupted in cancer patients, affecting the cell cycle, post-translational modifications, DNA replication and repair, and metabolic pathways [14,15,16,17]. Several rhythm genes have been found to be differentially expressed in HCC. The circadian clock regulators BMAL1 and CLOCK promote HCC cell proliferation by controlling Wee1 and p21 levels, thereby preventing apoptosis and cell cycle arrest [18]. Zheng et al. reported that the circadian gene CSNK1D enhances the Wnt/β-catenin pathway, promoting HCC progression [19]. In *p53* knockout mice, *Cry1* reduction enhances apoptotic sensitivity, reduces cancer risk, and extends lifespan, although this effect has not been confirmed in HCC [20]. CRY2 inhibits breast cancer cell proliferation, but its acetylation attenuates this antiproliferative effect [21]. Additionally, *Cry1* modulates chemoresistance in coordination with NANOG in cervical cancer patients [22]. In HCC, circadian clock genes also influence tumor immune cell infiltration [23]. This has led to the emergence of “biological clock therapy,” which integrates circadian rhythms into cancer treatment strategies [24]. Numerous preclinical and clinical studies have investigated this approach, yielding promising results [25]. A comprehensive understanding of the mechanisms underlying biological clock-based therapies may enhance their clinical application in cancer treatment.

In this study, bioinformatics analyses using multiple databases revealed that reduced expression of the circadian gene *Cry1* is associated with shorter overall survival in HCC patients. To investigate the role of *Cry1* in HCC progression, we modulated its expression through knockdown and overexpression *in vitro*. Our results show that *Cry1* significantly inhibits the invasion, migration, and proliferation of HCC cells. Moreover, *Cry1* suppresses the tumorigenicity of HCC cells, likely through the BCL2/BAX signaling pathway.

## 2 Methods

### 2.1 Bioinformatics analysis

To investigate the relationship between circadian rhythms and HCC, we performed bioinformatics analyses to identify relevant circadian genes in HCC patients using data from The Human Protein Atlas (https://www.proteinatlas.org). Kaplan–Meier Plotter (www.kmplot.com) was used to perform survival analysis in HCC patients.

### 2.2 Cell culture

The SMMC-7721 and LO2 cell lines were obtained from Procell Life Science & Technology Co., Ltd., and authenticated via 8-loci STR profiling. SMMC-7721 and LO2 cells were cultured in Dulbecco’s Modified Eagle’s Medium (Gibco) supplemented with 10% fetal bovine serum (Life Technologies), 100 μg/mL streptomycin, and 100 U/mL penicillin, in an incubator at 37°C with 5% CO_2_.

### 2.3 Cell transfection

1 × 10^6^ cells were incubated with lentivirus for *Cry1* overexpression or knockdown for 12 h at 37°C. A lentivirus lacking *Cry1* served as the negative control. After 48 h of incubation at 37°C, the medium was replaced with fresh culture medium, and successfully transduced cells were selected using puromycin (4 μg/mL). Transduction efficiency of approximately 70% was confirmed by GFP fluorescence analysis. Subsequently, stable cell lines were established: one overexpressing *Cry1* (SMMC-7721/*Cry1*) and its control (SMMC-7721/vehicle) and another with *Cry1* knockdown (SMMC-7721/sh*Cry1*) and its control (SMMC-7721/control).

### 2.4 Cell counting kit-8 (CCK-8) assay

Approximately 2,000 cells were seeded per well in 96-well plates. Cell viability was assessed at 24, 48, 72, and 96 h after seeding. At each time point, cells were incubated with CCK-8 reagent in serum-free medium for 2 h in the dark. Absorbance was measured at 450 nm using a Multiskan FC microplate reader (Thermo Fisher Scientific).

### 2.5 Wound healing assay

About 200,000 cells per well of 6-well plates were used to seed the cells. When the cells reached approximately 80% confluence, a straight scratch was made using a 200 μL sterile pipette tip. Detached cells were removed by washing with phosphate-buffered saline (PBS), and fresh serum-free medium was added. Images were captured at 0 and 48 h after scratching. Cell migration was quantified using the following formula: migration rate = [(0 h area – 48 h area)/0 h area] × 100%.

### 2.6 Transwell assay

A total of 0.5 × 10^5^ cells were seeded into the upper chamber of each Transwell insert. The lower chamber was filled with 600 μL of complete medium containing serum and incubated at 37°C for 24 h. After fixation with 4% paraformaldehyde for 15 min and staining with crystal violet for another 15 min, five random fields per well were imaged under a microscope at 100× magnification.

### 2.7 Apoptosis assay

Approximately 2.5 × 10^5^ cells were seeded per well in six-well plates. After 48 h of incubation, cells were harvested by trypsinization to obtain a single-cell suspension. The cell suspension was then incubated with 195 μL binding buffer with 5 μL Annexin V-FITC and 10 μL propidium iodide (PI). The mixture was incubated in the dark at room temperature for 20–30 min. After incubation, cells were filtered and analyzed by flow cytometry to assess apoptosis and cell viability.

### 2.8 Cell cycle assay

Approximately 2.5 × 10^5^ cells were seeded per well in six-well plates, then harvested by trypsinization and centrifuged to obtain a single-cell suspension. Cells were washed with pre-cooled PBS, fixed in 70% ethanol, and stored at 4°C for 24 h prior to staining with PI. After incubation with PI at 37°C for 30 min in the dark, cells were filtered and analyzed by flow cytometry for cell cycle distribution within 2 h.

### 2.9 RNA extraction and RT-qPCR

Total RNA was extracted from HCC cells using TRIzol reagent and then reverse-transcribed into cDNA. Quantitative real-time PCR (RT-qPCR) was performed using a one-step RT-qPCR SuperMix (TransGen Biotech, China). Relative mRNA expression levels were normalized to GAPDH using the 2^−ΔΔCt^ method. The primer sequences used are listed below:

*Cry1* Forward: GTGACAGCAGAGTCCCATGA

*Cry1* Reverse: CACTGCCATCTCGAGTTCAA

*Gapdh* Forward: ACTTTGGTATCGTGGAAGGACTCAT

*Gapdh* Reverse: GTTTTTCTAGACGGCAGGTCAGG.

### 2.10 Western blotting assay

Cells were lysed on ice using radioimmunoprecipitation assay buffer, and equal amounts of protein were separated on a 10% sodium dodecyl sulfate–polyacrylamide gel electrophoresis gel and transferred onto polyvinylidene fluoride membranes. Membranes were incubated with primary antibodies overnight at 4°C, followed by incubation with secondary antibodies for 2 h at room temperature. The following primary antibodies were used: BAX (ab182733, Abcam, 1:1,000), BCL2 (ab32124, Abcam, 1:1,000), and β-actin (60008-1-IG, Proteintech, 1:2,000). Protein bands were visualized using an ECL Enhanced Chemiluminescence Substrate Kit (Amersham) according to the manufacturer’s instructions.

### 2.11 Colony formation assay

A total of 1,000 cells were seeded per well in 6-well plates and cultured for 2 weeks. The cells were fixed with 4% paraformaldehyde for 20 min and stained with crystal violet for 15 min. Colonies containing more than 50 cells were considered positive for colony formation.

### 2.12 Statistical analysis

All experiments were performed in triplicate or more. Two-tailed unpaired Student’s *t*-test was used to analyse the Statistical differences between the two groups. Differences were considered statistically significant at *P* < 0.05 (*P < 0.05, **P < 0.01, ***P < 0.001).

## 3 Results

### 3.1 Low expression of *Cry1* was associated with shorter overall survival in HCC patients

**Figure 1:**
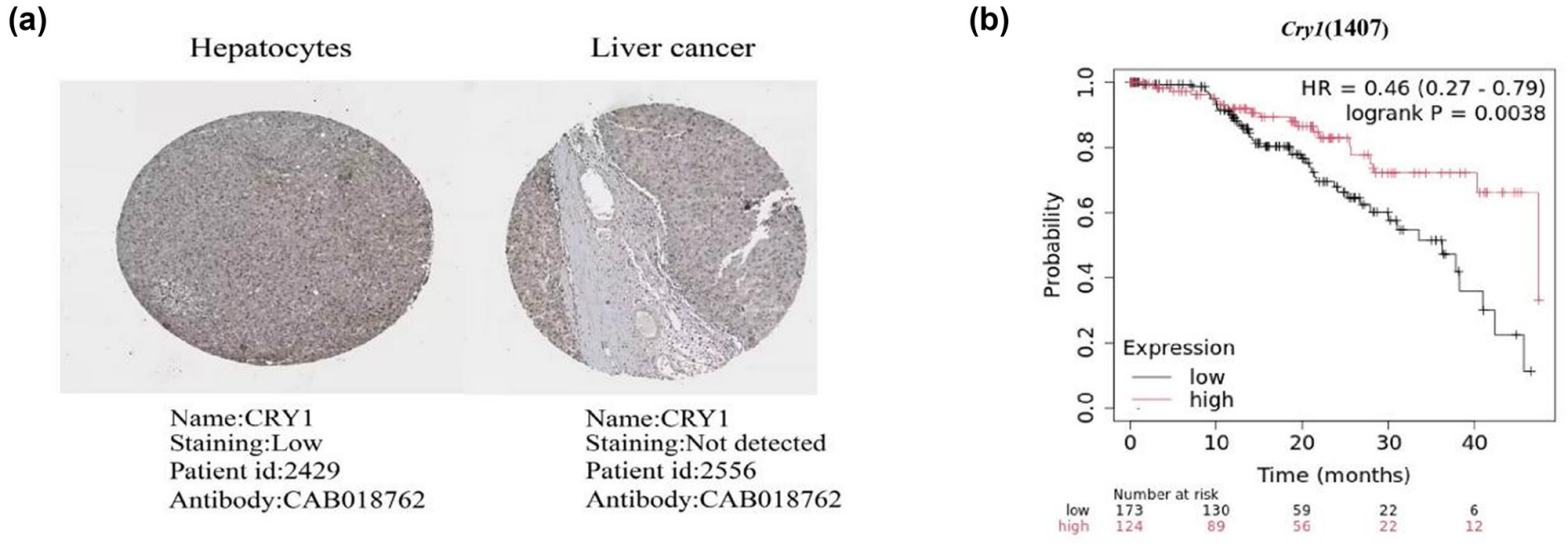
*Cry1* expression was associated with HCC. (a) Immunohistochemical staining of *Cry1* protein levels: normal tissue is shown on the left and cancer tissue on the right. (b) Survival analysis plot based on *Cry1* expression. Log-rank test was used to analyse the statistical significance. Statistical significance: ***P < 0.001, **P < 0.01, *P < 0.05.

To investigate the association between circadian rhythm and HCC, bioinformatics analysis was performed to identify relevant rhythm genes in HCC patients using data from the Human Protein Atlas (https://www.proteinatlas.org). Analysis of immunohistochemical staining data revealed that HCC tissues exhibited significantly lower levels of CRY1 protein compared to normal tissues (Figure 1a). Subsequent analysis using the KM plotter online tool showed that low *Cry1* expression was significantly associated with shorter overall survival in HCC patients (Figure 1b). These findings indicate that *Cry1* expression is closely associated with HCC.

### 3.2 *Cry1* expression was downregulated and exhibited rhythmicity in HCC cells

**Figure 2:**
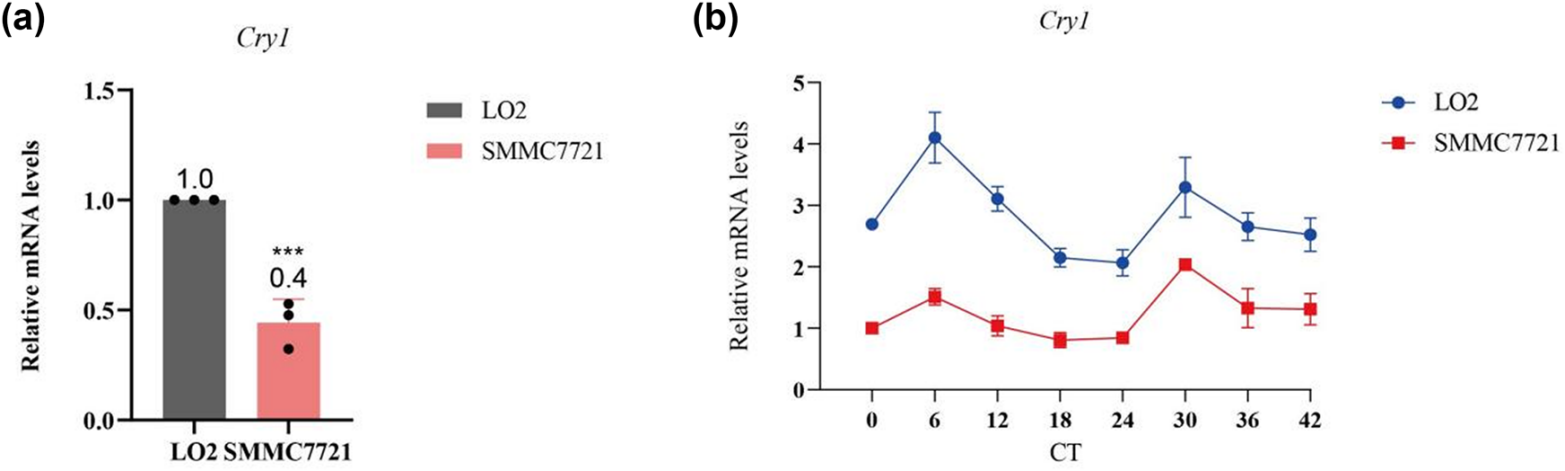
*Cry1* expression in HCC cells. (a) Quantitative analysis of *Cry1* mRNA expression in SMMC-7721 cells by RT-qPCR. (b) Rhythmic oscillation of *Cry1* in SMMC-7721 cells. Statistical significance: ***P < 0.001, **P < 0.01, *P < 0.05.

To further explore the relationship between *Cry1* and HCC progression, *Cry1* expression was analyzed in HCC cells (SMMC-7721). Dexamethasone(0.05 mg/mL) treatment was applied for 2 h to synchronize SMMC-7721 cells and normal hepatocytes (LO2). Cells were harvested every 6 h from CT0 to CT42, and total RNA was extracted at each time point (CT0, CT6, CT12, CT18, CT24, CT30, CT36, and CT42). RT-qPCR results demonstrated that *Cry1* expression was reduced in SMMC-7721 cells (Figure 2a). Furthermore, *Cry1* exhibited rhythmic expression in both SMMC-7721 cells and normal hepatocytes, peaking at CT6 and reaching a trough at CT18 (Figure 2b). Moreover, the peak-to-trough amplitude was about 2-fold in normal hepatocytes (LO2) and about 1.5-fold in SMMC-7721 cells.

### 3.3 SMMC-7721/sh*Cry1* and SMMC-7721/*Cry1* cell lines were generated

**Figure 3:**
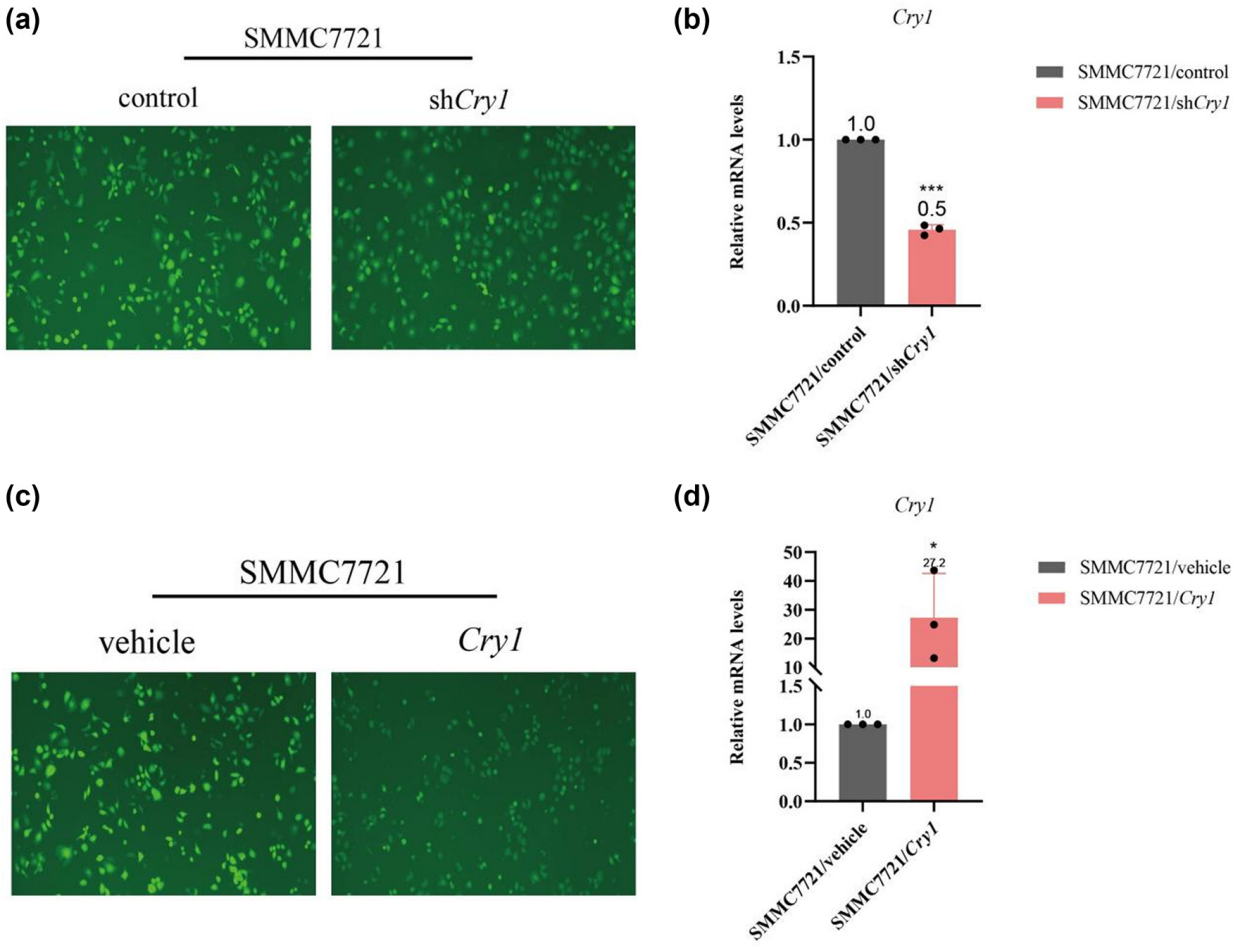
*Cry1* mRNA levels in stably transfected HCC cell lines. (a) Green fluorescence images of *Cry1*-transfected SMMC-7721 cells showing knockdown and control groups. (b) RT-qPCR analysis of *Cry1* mRNA levels in SMMC-7721/sh*Cry1* cells. (c) Green fluorescence images of *Cry1*-transfected SMMC-7721 cells showing overexpression and control groups. (d) RT-qPCR analysis of *Cry1* mRNA levels in SMMC-7721/*Cry1* cells. “sh*Cry1*” denotes the knockdown group and “Control” the corresponding control; “*Cry1*” denotes the overexpression group and “Vehicle” its control. These descriptions will not be repeated in subsequent text. Statistical significance: ***P < 0.001, **P < 0.01, *P < 0.05.

To investigate the role of *Cry1* in HCC, *Cry1* expression was either knocked down or overexpressed in SMMC-7721 cells via lentiviral transfection using siRNA knockdown and overexpression constructs. RT-qPCR results demonstrated that *Cry1* mRNA levels were significantly decreased in SMMC-7721/sh*Cry1* stable knockdown cell lines (Figure 3a and b) and increased in SMMC-7721/*Cry1* stable overexpression cell lines (Figure 3c and d). These results confirmed successful knockdown or overexpression of *Cry1* in SMMC-7721 cells.

### 3.4 *Cry1* inhibits the proliferation of HCC cells

**Figure 4:**
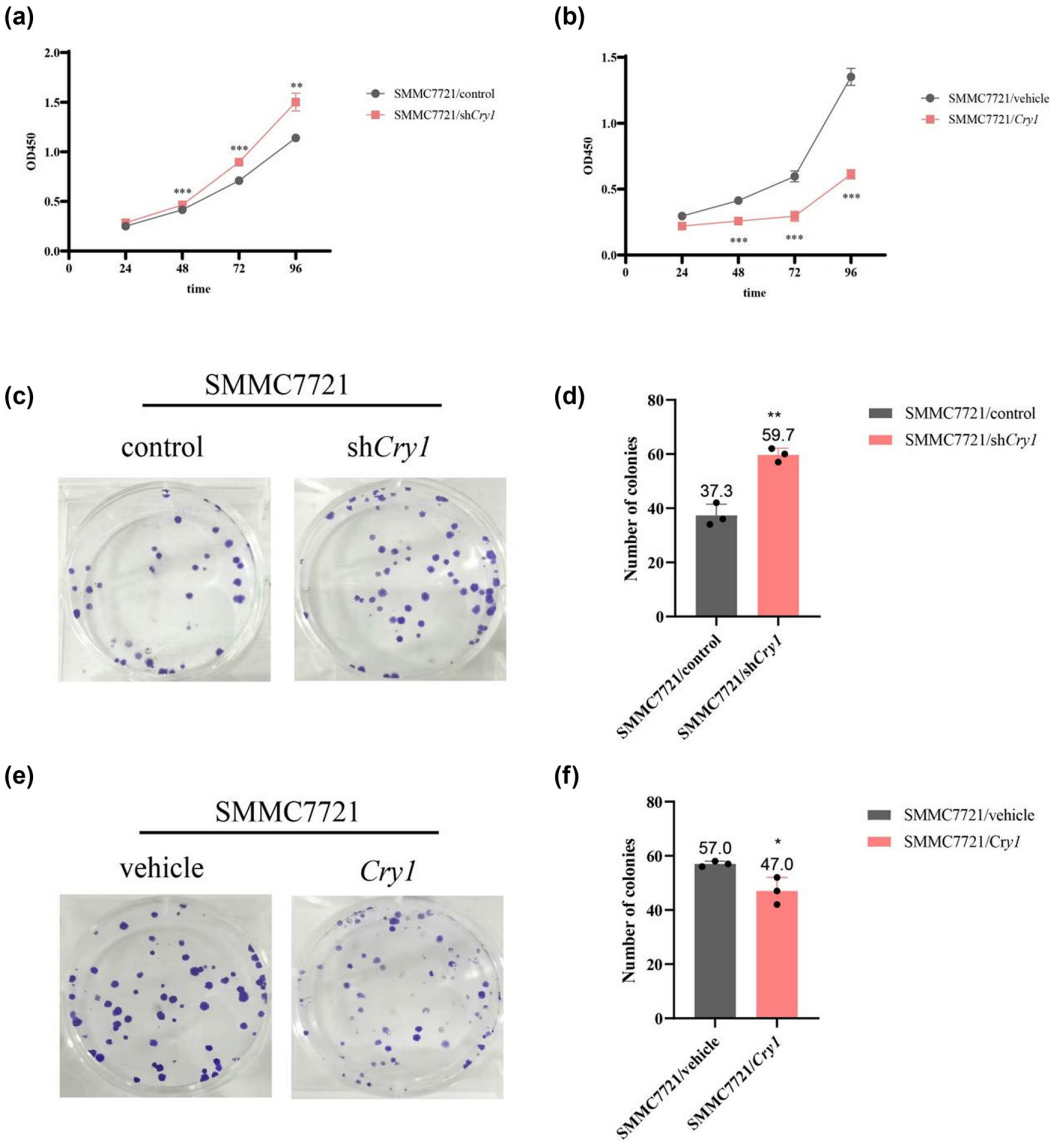
*Cry1* inhibits the proliferation of HCC cells. (a and b) Effects of *Cry1* knockdown and overexpression on SMMC-7721 cell proliferation were evaluated using the CCK-8 assay. (c and d) Effects of *Cry1* knockdown on colony formation of SMMC-7721 cells were assessed after 2 weeks. (e and f) Effects of *Cry1* overexpression on colony formation of SMMC-7721 cells were assessed after 2 weeks. Statistical significance: ***P < 0.001, **P < 0.01, *P < 0.05.

To assess the effect of *Cry1* on HCC cell proliferation, CCK-8 and colony formation assays were performed. Results showed that *Cry1* knockdown (SMMC-7721/sh*Cry1*) significantly increased SMMC-7721 cell proliferation (Figure 4a), whereas *Cry1* overexpression (SMMC-7721/*Cry1*) significantly suppressed proliferation (Figure 4b). To further examine the effect of *Cry1* on proliferation, a colony formation assay was conducted. The number of colonies formed following *Cry1* knockdown was 59.7 ± 0.07, significantly higher than the control group. *Cry1* knockdown thus promotes colony formation (Figure 4c and d), whereas *Cry1* overexpression resulted in 47 ± 0.27 colonies, significantly fewer than controls, indicating that *Cry1* overexpression inhibits colony formation (Figure 4e and f). These results indicate that *Cry1* inhibits HCC cell proliferation.

### 3.5 *Cry1* induced G1 phase arrest in HCC cells

**Figure 5:**
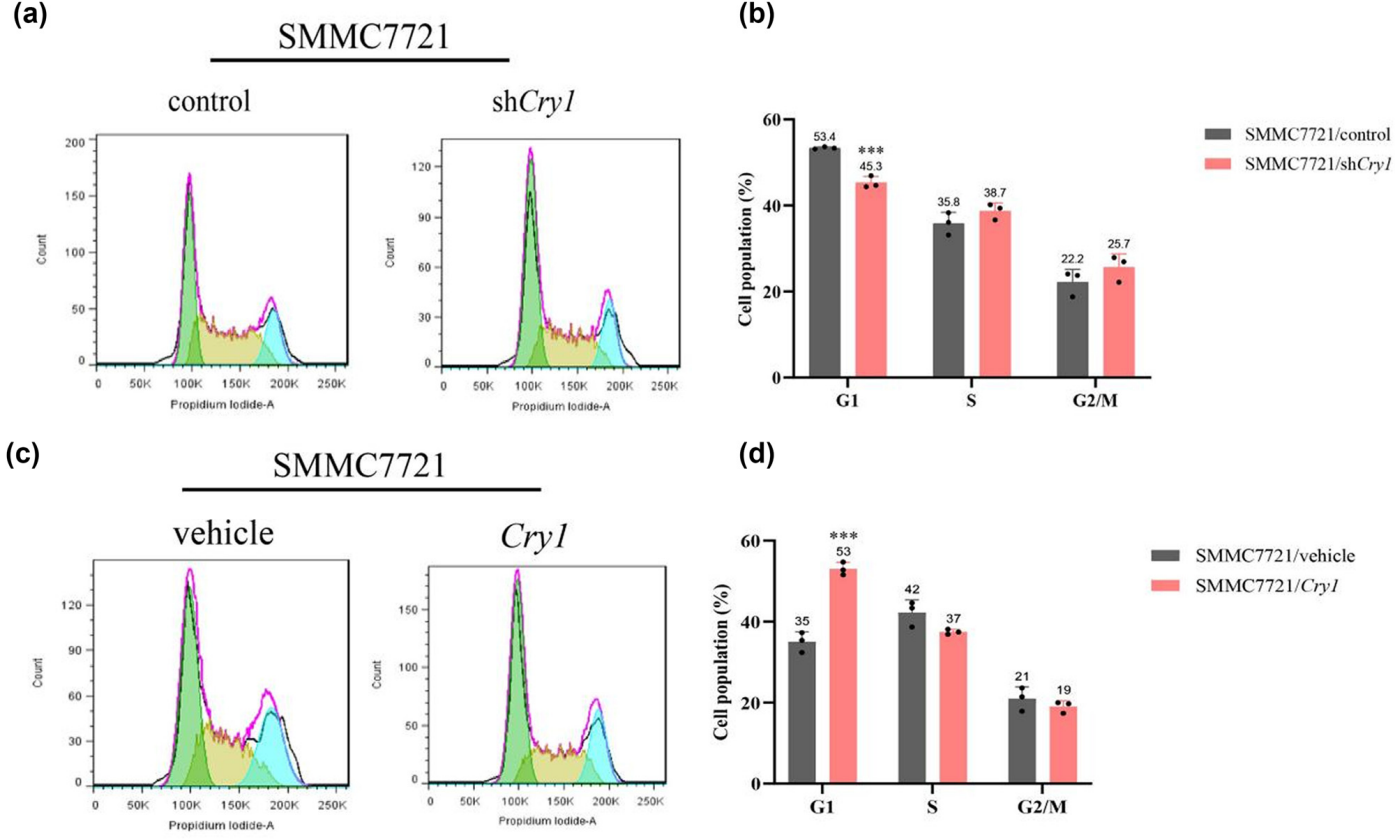
*Cry1* influenced the cell cycle distribution of HCC cells. (a and b) Quantitative analysis of cell cycle phases in SMMC-7721/sh*Cry1* and SMMC-7721/control cells by flow cytometry. (c and d) Quantitative analysis of cell cycle phases in SMMC-7721/*Cry1* and SMMC-7721/vehicle cells by flow cytometry. Statistical significance: ***P < 0.001, **P < 0.01, *P < 0.05.

To further explore the effect of *Cry1* on HCC cell proliferation, flow cytometry analysis was performed. Cell cycle distribution of SMMC-7721 cells was analyzed, revealing that *Cry1* knockdown significantly decreased the proportion of cells in the G1 phase, 45.3% in sh*Cry1* vs 53.4% in control (Figure 5a and b), whereas *Cry1* overexpression markedly increased the G1 phase population, 53% in *Cry1* vs 35% in control (Figure 5c and d). These findings indicate that *Cry1* induces G1 phase arrest in HCC cells, thereby inhibiting their proliferation.

### 3.6 *Cry1* inhibits the migratory ability of HCC cells

**Figure 6:**
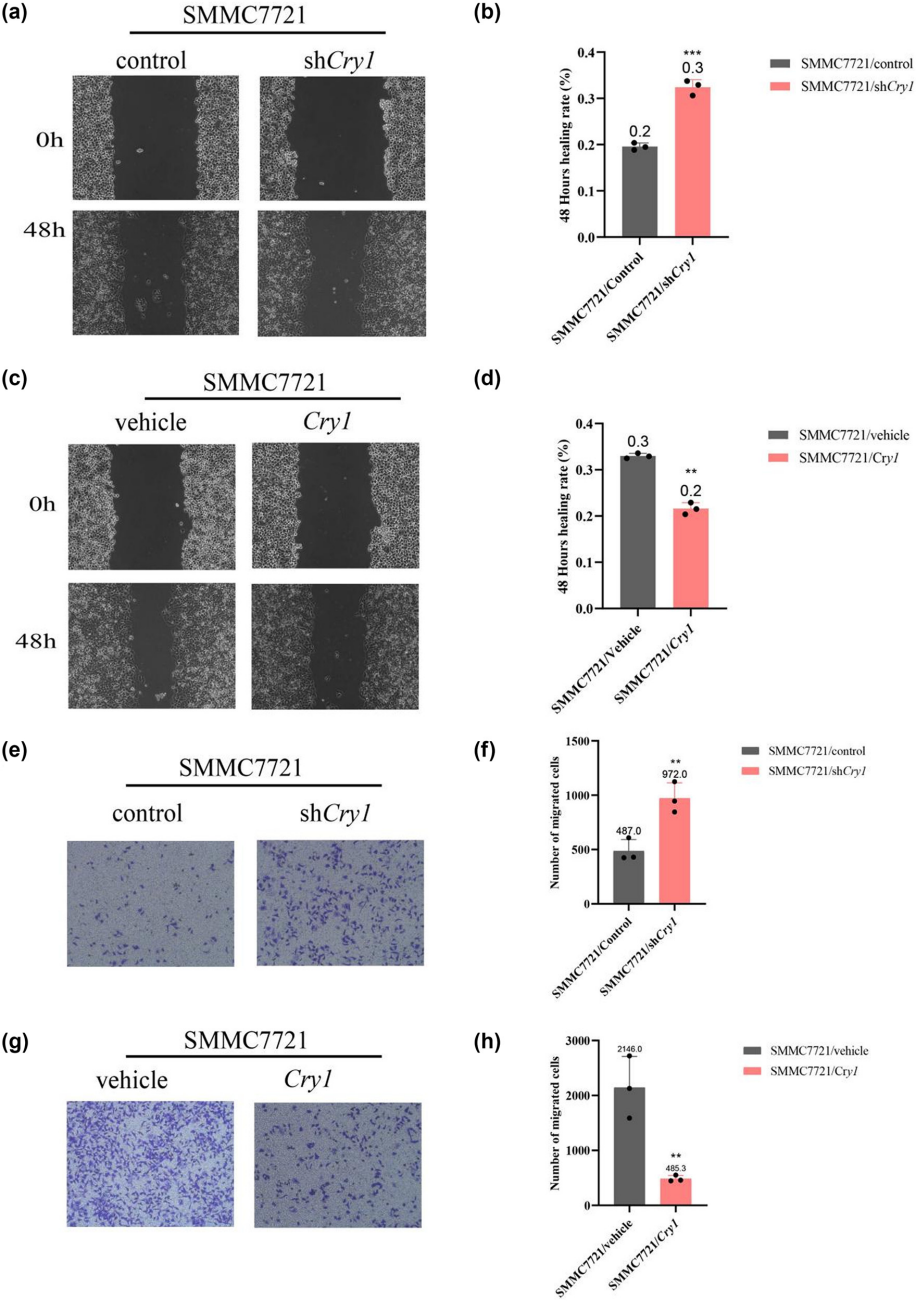
*Cry1* inhibits the migratory ability of HCC cells. (a and b) Wound healing assays evaluated the effect of *Cry1* knockdown on SMMC-7721 cell migration. (c and d) Wound healing assays evaluated the effect of *Cry1* overexpression on SMMC-7721 cell migration. (e and f) Transwell migration assays assessed the impact of *Cry1* knockdown on SMMC-7721 cell migration. (g and h) Transwell migration assays assessed the impact of *Cry1* overexpression on SMMC-7721 cell migration. Statistical significance: ***P < 0.001, **P < 0.01, *P < 0.05.

To assess the effect of *Cry1* on HCC cell migration, wound healing and transwell assays were performed. Wound healing assays demonstrated that *Cry1* knockdown enhanced wound closure and cell migration (Figure 6a and b), whereas *Cry1* overexpression significantly inhibited these processes (Figure 6c and d). Consistent with wound healing results, transwell assays revealed a significant increase in the number of migrated SMMC-7721 cells following *Cry1* knockdown (Figure 6e and f), whereas *Cry1* overexpression markedly reduced cell migration (Figure 6g and h). These findings suggest that *Cry1* inhibits the migration of HCC cells.

### 3.7 *Cry1* inhibits the invasive ability of HCC cells

**Figure 7:**
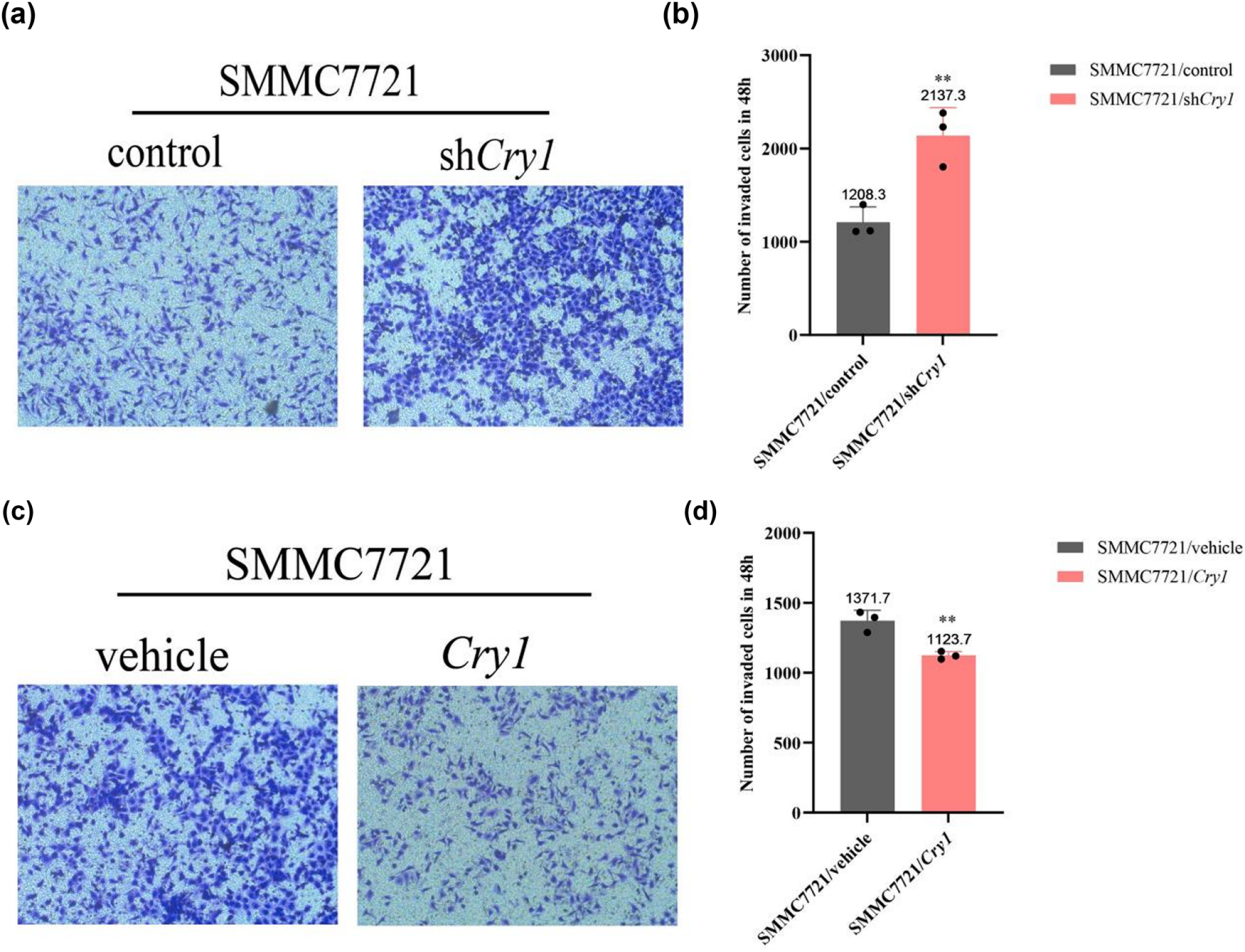
*Cry1* inhibits the invasive ability of HCC cells. (a and b) The effects of *Cry1* knockdown on SMMC-7721 cell invasion were assessed using Matrigel-coated Transwell assays. (c and d) The effects of *Cry1* overexpression on SMMC-7721 cell invasion were assessed using Matrigel-coated Transwell assays. Statistical significance: ***P < 0.001, **P < 0.01, *P < 0.05.

To assess the effect of *Cry1* on HCC cell invasion, Transwell invasion assays were performed. Results demonstrated that *Cry1* knockdown increased the number of invading SMMC-7721 cells, 2137.3 in sh*Cry1* vs 1208.3 in control (Figure 7a and b), whereas *Cry1* overexpression decreased the number of invading cells, 1123.7 in *Cry1* vs 1371.7 in control (Figure 7c and d). These results indicate that *Cry1* inhibits HCC cell invasion.

### 3.8 *Cry1* promotes apoptosis in HCC cells by modulating the expression of apoptosis-related proteins

**Figure 8:**
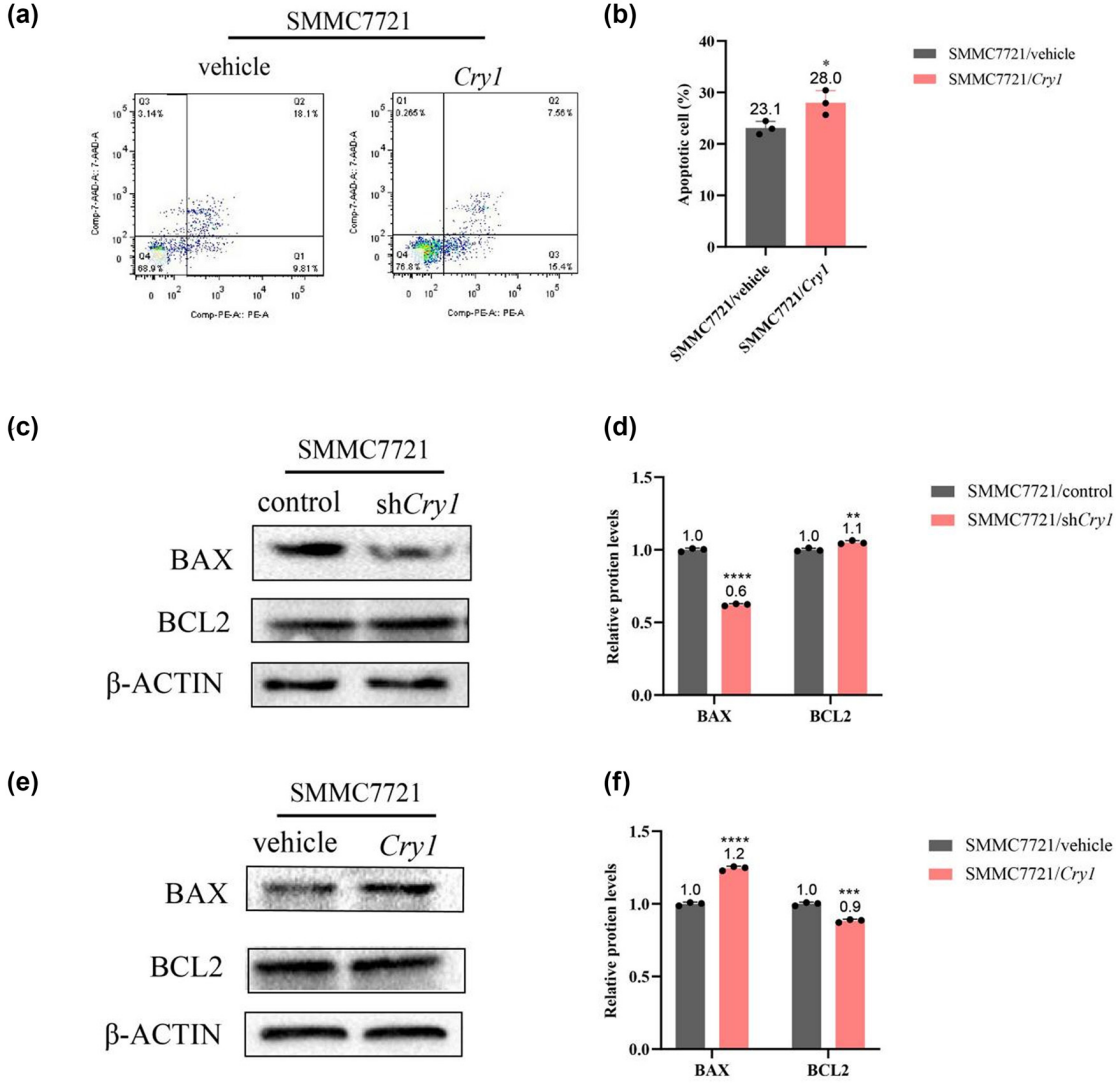
*Cry1* promotes apoptosis in HCC cells by modulating the expression of apoptosis-related proteins. (a and b) Effect of *Cry1* overexpression on apoptosis in HCC cells as determined by flow cytometry. (c and d) Effect of *Cry1* knockdown on the expression of apoptosis-related proteins in HCC cells analyzed by Western blotting. (e and f) Effect of *Cry1* overexpression on the expression of apoptosis-related proteins in HCC cells analyzed by Western blotting. Statistical significance: ****P < 0.0001, ***P < 0.001, **P < 0.01, *P < 0.05.

To evaluate the effect of *Cry1* on apoptosis in HCC cells, flow cytometry was performed. *Cry1* overexpression significantly increased apoptosis in SMMC-7721 cells compared to control cells, 28% in *Cry1* vs 23.1% in control (Figure 8a and b). Flow cytometry results indicated that *Cry1* promotes apoptosis in HCC cells. To further investigate the molecular mechanism underlying *Cry1*-induced apoptosis, the protein levels of apoptosis-related genes were examined. Western blot analysis revealed that *Cry1* knockdown reduced the expression of the pro-apoptotic protein BAX (0.6-fold lower) and increased the expression of the anti-apoptotic protein BCL2 (Figure 8c and d), whereas *Cry1* overexpression upregulated BAX (1.2-fold increased) and downregulated BCL2 (Figure 8e and f). These results suggest that *Cry1* promotes apoptosis in HCC cells through the BCL2/BAX-mediated signaling pathway.

## 4 Discussion

Disruption of the circadian rhythm can lead to a range of health issues, including sleep disorders, hormonal imbalances, impaired immune function, accelerated aging, carcinogenesis, and neuropsychiatric conditions [26]. Cancer remains the leading cause of mortality worldwide and continues to be a major focus of biomedical research. A well-established association exists between circadian rhythm disruption and cancer, as the high prevalence of circadian abnormalities among cancer patients suggests a potential causal relationship [25,27]. Epidemiological studies have shown that women working long-term night shifts have a significantly increased risk of developing colorectal, breast, and endometrial cancers [28,29,30]. Similarly, male pilots frequently operating across time zones exhibit a higher risk of developing acute myeloid leukemia and prostate cancer compared to non-shift-working counterparts [31]. Furthermore, circadian clocks regulate key cellular processes, including apoptosis, DNA damage repair, the cell cycle, and energy metabolism [17]. These findings highlight the pivotal role of circadian clocks in tumor initiation and progression. In light of this evidence, cancer therapies are increasingly incorporating circadian timing, giving rise to the concept of chronotherapy [24]. A deeper understanding of circadian clock function and its underlying mechanisms in cancer treatment may enhance therapeutic strategies and improve patient outcomes.

Numerous studies have demonstrated that circadian genes play essential roles in cancer progression. Mice with *PER2* gene mutations exhibited a tenfold higher risk of developing lymphoma following ionizing radiation compared to wild-type mice [32]. The microRNA miR-3187-3p enhances cell invasion and migration in head and neck squamous cell carcinoma by targeting PER2 [33]. Deletion of the circadian gene *Per1* protects mice from severe ethanol-induced hepatotoxicity [34]. In glioma cells and familial breast tumors, *Per1* and *Per2* expression levels were significantly reduced compared to normal cells or tissues [35,36]. The *PER3* gene, potentially regulated by MEK/ERK signaling, functions as a tumor suppressor in breast cancer initiation and progression [37]. Emerging evidence demonstrates that CRY is critically involved in cancer pathogenesis, while our findings reveal its tumor-suppressive function through inhibiting HCC cell proliferation. Gul et al. identified M47 as a selective CRY1 degrader that potentiates oxaliplatin-induced apoptosis in Ras-transformed p53-null fibroblasts and extends median survival in p53−/− mice by approximately 25% [20]. Xia et al. demonstrated that Cry2 suppresses the proliferation of breast cancer cells by repressing genes within the NF-κB pathway, whereas its acetylation attenuates this inhibitory effect [21]. In cervical cancer, *Cry1* regulates chemoresistance by inhibiting apoptosis through the STAT3 pathway [22].

Moreover, artificial manipulation of circadian clock-associated genes has been found to significantly affect tumor development. For example, BMAL1 overexpression in ovarian cancer cell lines reduced tumor growth and restored c-MYC rhythmicity [38]. Similarly, in colorectal cancer cell lines, BMAL1 overexpression yielded comparable effects, and increased BMAL1 expression was associated with prolonged patient survival [39]. These findings suggest that circadian clock genes not only influence cancer initiation but also modulate its progression through gene expression regulation.

Chronotherapeutic approaches in cancer treatment are increasingly regarded as innovative and evidence-based strategies. These strategies involve administering anticancer agents at optimal times to align with circadian rhythms that regulate both therapeutic efficacy and drug-related toxicity in healthy tissues. Timing the administration of anticancer medications can enhance their therapeutic index while minimizing adverse effects [40]. Chronomodulated chemotherapy has been clinically applied in patients with advanced gastrointestinal cancer, yielding favorable outcomes [41]. A recent study reported that glioblastoma patients who received temozolomide in the morning exhibited improved overall survival [42]. According to Kireeva et al., the DNA damage responses triggered by cisplatin are indeed governed by circadian control exclusively in clock-proficient cells, which bears potential implications for enhancing or devising chronotherapy approaches for cancer patients [43]. Clinical evidence demonstrates that chronomodulated chemotherapy converts initially unresectable colorectal cancer liver metastases to resectable status, achieving 5-year survival rates of 39–50% in clinical cohorts [41]. Time-specific drug administration is proposed to be superior to conventional treatment by increasing efficacy and reducing side effects [38]. Our findings indicate that *Cry1* expression in HCC cells is both reduced and rhythmic, peaking at CT6 (Figure 2). While our results nominate *Cry1* as a candidate HCC target and suggest CT6 timing optimizes therapy, future preclinical studies are essential to validate these effects.

An imbalance between apoptosis and abnormal cell proliferation contributes to tumor development. Our findings revealed that *Cry1* overexpression inhibited HCC cell proliferation, whereas *Cry1* knockdown promoted it (Figure 4). *Cry1* functions as a tumor-specific regulator of DNA repair, controlling the G2/M transition in prostate cancer [44]. In contrast, our results showed that *Cry1* arrested the HCC cell cycle at the G1 phase (Figure 5), possibly due to the context-dependent role in HCC versus prostate cancer. The intrinsic apoptotic pathway is regulated by BCL2 family proteins, particularly the ratio of pro-apoptotic BAX to anti-apoptotic BCL2 [45]. BCL2 family proteins modulate outer mitochondrial membrane permeabilization, leading to the release of caspase activators into the cytosol and ultimately triggering cell death [46]. In diabetic cardiomyopathy, *BMAL1* overexpression enhanced BMAL1/BCL2 binding, which suppressed IP3R activity, reduced mitochondrial Ca^2+^ overload, and attenuated subsequent apoptosis [47]. The circadian gene *mPer2* is essential for tumor suppression in mice, promoting apoptosis by upregulating *p53* and *Bax* and downregulating *c-Myc*, *Bcl-xL*, and *Bcl2* [48]. Our previous study demonstrated that *Per3* promotes astroblastoma progression via the p53/BCL2/BAX signaling pathway [35]. In this study, we found that *Cry1* upregulates pro-apoptotic BAX and downregulates anti-apoptotic BCL2 in HCC cells (Figure 8).

Collectively, our results suggest that *Cry1* acts as a negative regulator of HCC tumorigenicity by modulating the apoptotic proteins BCL2 and BAX. However, the underlying mechanism by which *Cry1* regulates these apoptotic proteins remains unclear, likely via transcriptional repression of BCL2. Further studies are needed to elucidate this regulatory mechanism.

## 5 Conclusion

**Figure 9:**
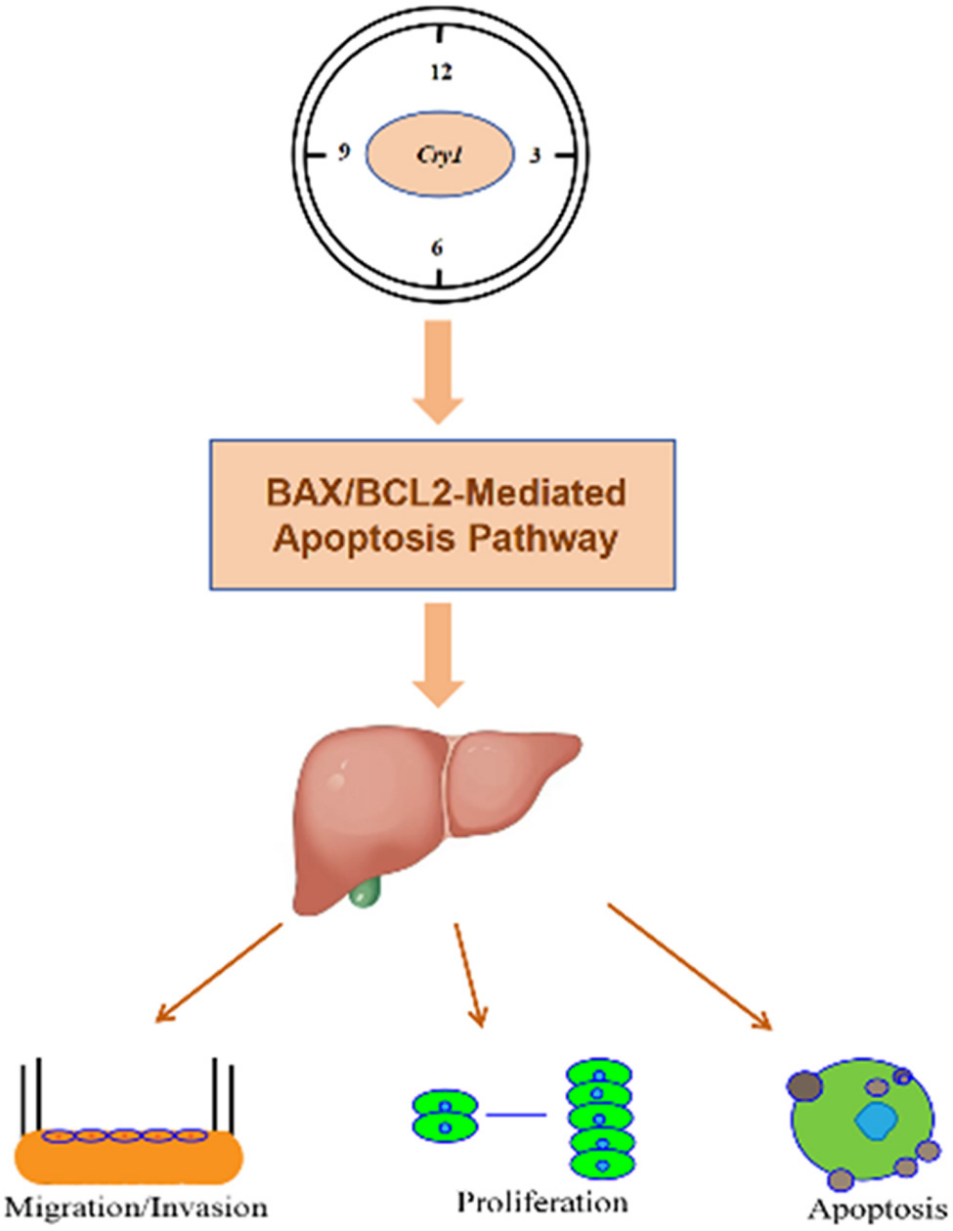
The working hypothesis of *Cry1* suppresses HCC progression via BCL2/BAX-mediated apoptosis pathway.

In summary, we found that *Cry1* expression in HCC cells was rhythmic, with a 24 h period peaking at CT6 and trough at CT18, but overall reduced compared to normal hepatocytes. *Cry1* may serve as a potential therapeutic target in liver cancer, as it appears to suppress HCC progression via increased BCL2/BAX ratio to promote apoptosis (Figure 9). However, its therapeutic potential requires further clinical validation.
